# Moving stereotactic fiducial system to obtain a respiratory signal: proof of principle

**DOI:** 10.1120/jacmp.v17i1.5633

**Published:** 2016-01-08

**Authors:** Roberto Caballero Pinelo, Rodolfo Alfonso, Yelina González Pérez, Albin Ariel García, Arnaldo Rubio

**Affiliations:** ^1^ Department of Radiation Oncology Hermanos Ameijeiras Hospital (HHA) Havana Cuba; ^2^ Nuclear Engineering Department Institute of Applied Sciences and Technologies (INSTEC) Havana Cuba

**Keywords:** 4D CT, respiratory signal, stereotactic body frame, cine CT

## Abstract

The purpose of this study was to obtain a respiratory signal with the use of an add‐on device to a specific stereotactic body frame and evaluate precision and accuracy of the method, with the use of a dynamic phantom. The authors designed and constructed a simple add‐on device which, attached to a stereotactic body frame, provides information of the patient's respiratory signal in every CT axial image acquired. To assess the approach, 12 CT studies were acquired, on a phantom that simulates respiratory motion, which was placed inside the frame with the add‐on device. Images of the phantom with sinusoidal and shark‐fin motion patterns were acquired, with different amplitude in the movement of the external surrogate and the target. Cycle time was 6 s. Images were retrospectively processed to obtain a respiratory signal from the vertical movement of the “abdomen.” The obtained signal was adjusted to a sinusoidal function; the resultant amplitude and cycle time were compared with the preset function in the phantom. The cycle amplitude and time obtained with the method agreed with the preset values within 0.4 mm and 0.29 s, respectively. In the cases of sinusoidal movements the maximal discrepancy was less than 1 mm. A respiratory signal was obtained in all cine CT sequence studies with this method that consistently coincides with the preset motion of the phantom. The authors proposed a tool to obtain a respiratory signal based on information contained into the CT axial images.

PACS number: 87.57qp

## I. INTRODUCTION

The management of the respiratory motion in tomographic images with the purpose of planning radiation therapy has become important.^1^, ^2^ In radiation therapy, this motion has begun to be quantified for treatments of stereotactic body and conventional radiation therapy when treating abdominal and thoracic tumors. The information and images obtained are used to view the entire range of tumor motion throughout the breathing cycle and adapt the treatment to these motion displacements.

For this purpose, 4D computed tomography (4D CT) plays a very important role. Methods developed over the past decade^3^, ^4^, ^5^, ^6^ apply direct and indirect approaches. The direct approach method employs implanted fiducial and kV or MV images to track the tumor location.^(7)^ Indirect approaches employ either a single or multiple external surrogates, or measure the tidal volume of patient respiration. The tumor location is determined based either on the correlation between the external surrogate and internal organs or on the correlation between the tidal volume and internal organs.^(7)^

In low/middle income countries (LMIC), radiation therapy institutions count only a few CT scanners due to scarce funding. These scanners are usually not of the latest technology, meaning that the standard procedures recommended in international guidelines need to be adapted to the local conditions. As our institution is located in an LMIC, radiotherapy services' access to scanners with 4D CT capabilities is limited. So one of the purposes of this paper is to design and test a simple, low‐cost methodology for acquiring CT images that consider the motion of the organs, using CT scanners with no high‐end features. Moreover, only an older machine was available to perform the tests reported in this paper. These CTs can perform a subsecond scan, but there is usually no access to the devices and licenses needed to acquire a 4D CT study. The cost of commercial respiratory motion management systems for simulation can exceed $50K,^7^, ^8^ so the use of respiratory‐guided techniques is limited.

This work uses the stereotactic body frame (SBF) from Elekta (Elekta Instrument AB, Stockholm, Sweden), conceived for immobilization and a precise localization of the tumor guided by a stereotactic fiducial system.

Historically, in order to minimize the variations in the localization of the tumors in the abdomen and thorax, the developers of SBRT^(1)^ scanned the patient in a body frame with a built‐in coordinate system that could be visualized in the CT image. The current availability of IGRT has made this older body frame/fiducial‐based system obsolete. Soft‐tissue targets require volumetric imaging such as CBCT or CT‐on‐rails to achieve the necessary setup precision.^(1)^ Treatment machines with gantry‐mounted kV units capable of fluoroscopy, radiographic localization, and cone‐beam imaging, especially for soft‐tissue targets, are being widely adopted. This has had a profound effect on SBRT.

Although manufacturers and AAPM TG‐101^(1)^ recommend limiting old fiducial stereotactic body frames to immobilization and first‐order localization, to be refined later using IGRT techniques, many facilities use these devices without an IGRT system.

This work describes the design and construction of a device that is used as an add‐on to the Elekta stereotactic body frame, that permits obtaining a respiratory signal and creates conditions for obtaining CT images in different respiratory phases in a patient or a phantom.

This device and procedure are simple and low‐cost. The idea can be adapted to a different frame system with small changes, and therefore can be utilized in almost every department with a multislice CT.

## II. MATERIALS AND METHODS

### A. Stereotactic body frame SBF, systems description

The stereotactic body frame (SBF) created and developed by the Karolinska Hospital group, Stockholm, Sweden,^(9)^ and since the mid‐1990s marketed by Elekta, is used in this work.

**Figure 1:**
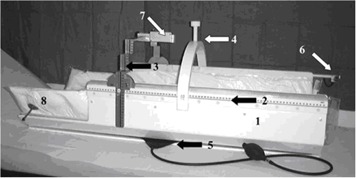
The Elekta stereotactic body frame (SBF). (1) Sidewall containing oblique and horizontal copper wires for CT‐based measurement of longitudinal stereotactic coordinate, the same system is available with copper sulfate fill for use in MRI. (2) Longitudinal stereotactic scale. (3) Stereotactic arc for lateral and AP coordinates. (4) Arc and scaled screw for diaphragm control. (5) Level control. (6,7) SBF attached laser system (leg and trunk) for assistance at patient repositioning. (8) Vacuum pillow. (Taken from Wulf^(9)^)

The technical concept of the SBF, displayed in Fig. 1, addresses three basic requirements for extracranial stereotactic radiotherapy:

1. Patient fixation,
2. An external reference system for determination, localization, and alignment of the stereotactic coordinates, and
3. A mechanical tool for reduction of breathing mobility.

The SBF locates the target volume using both CT (computed tomography) and MR (magnetic resonance).

A detailed description of the SBF can be found in Wulf^(9)^ and Slotman, et al.^(10)^

### B. Add‐on device

**Figure 2:**
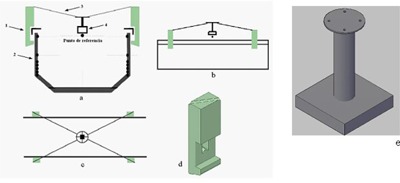
Device designed to obtain the respiratory signal in the CT images. Front view (a) shows: 1, piece to couple on the lateral of the SBF; 2, SBF; 3, CT‐compatible rod; 4, central piece, to be placed on patient abdomen between the umbilicus and the xifoid process. Lateral view (b). Superior view (c). (d) Shows piece to couple on the lateral of the SBF; (e) shows central piece.

The respiratory signal will be obtained with the information contained in the CT images with the use of an add‐on device compatible with the CT image and suitable to the SBF system. Figure 2 show the components of the device.

**Figure 3:**
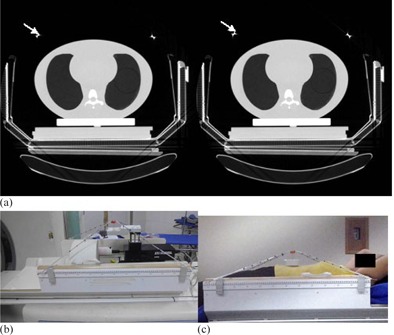
CT images (a) of a cine sequence to appreciate the variation on the same slice position of the Y coordinate of the center of the wires (white arrows). In the picture on the right the wires are in a higher position. The CIRS phantom (b) placed inside the SBF with the add‐on device to detect the respiratory signal. A volunteer on the SBF (c) with the add‐on device.

The system consists of four narrow, CT‐compatible bars, a central piece and four pieces to be attached to the lateral walls of the SBF. The CT‐compatible bars were made of acrylic (PMMA), hollow inside and in the center a copper wire, which is seen on every axial CT image with high intensity in the portion that intersects the plane of the image (Fig. 3).

The bars used in this work were 470 mm long, hollow inside, 5 mm external diameter and 3 mm internal diameter, and inside the bars a copper wire of 2 mm diameter was introduced, which is visible on the images as a high‐density signal. Windowing the images and processing them, the coordinates of the bars section center seen on the images could be identified automatically. These images were processed with ImageJ2x software (freeware available at http://rsb.info.nih.gov/ij/).

When the patient or phantom is “breathing,” the bars are fixed to the upper portion of the central piece and they slide on the grooves cut on the pieces that couple with the lateral walls of the SBF. As a prototype, these lateral pieces and the base of the central piece were made of Styrofoam available in the molding room of the department and shaped with the block cutter.

### C. Dynamic thorax phantom

The Computerized Imaging Reference Systems (CIRS) Dynamic Thorax Phantom model 008A (CIRS, Norfolk, VA) with CIRS motion control software was used in this work.

The CIRS Dynamic Thorax Phantom is a precision instrument for investigating and minimizing the impact of tumor motion inside the lung. It provides known, accurate, and repeatable three‐dimensional target motion inside a tissue‐equivalent phantom. It is designed for comprehensive analysis of image acquisition, planning, and dose delivery in image‐guided radiation therapy.

**Figure 4:**
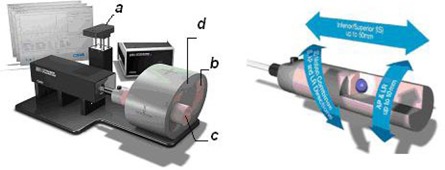
The CIRS 008A phantom: (a) surrogate, (b) lung equivalent lobe, (c) lung equivalent rod containing a spherical target, (d) muscle equivalent density.

The phantom body represents an average human thorax in shape, proportion, and composition, but without a rib cage (Fig. 4). It has a surrogate (a) that represents the movement of the abdomen, a lung‐equivalent lobe (b), and the corresponding sections with muscle‐equivalent density (d). A lung‐equivalent rod containing a spherical target (c) and/or various detectors is/are inserted into the lung‐equivalent lobe of the phantom. The rod is connected to a motion actuator box that induces three‐dimensional target motion through linear translation and rotation of the lung‐equivalent rod. Motion of the rod itself is radiographically invisible due to its matching density with the surrounding material. The target and its motion, given its density difference, can be resolved. Manufacturer reports a motion accuracy of 0.1 mm.

Target and surrogate motion can be independently controlled with CIRS motion control software. Amplitude, cycle time, and phase shift can be applied to both the surrogate and main phantom.

In this work we used the rod with spherical targets of 1 and 3 cm diameter.

The phantom was scanned on a Philips Mx8000 10‐slice CT scanner (Philips Healthcare, Andover, MD). Cine mode sequential images were acquired with a slab of 2.4 cm width and eight slices, 3 mm slice thickness. Each slab was acquired with an exposure time of 0.5 s, and time between each image of ∼0.8 s, cine acquisition duration was 16 s, other parameters were 120 kVp, 100 mAs, filter B, matrix scan 768×768, with ultrafast resolution image, and FOV 500 mm

A protocol was programmed in the CT console called 4D CT with four contiguous slabs. If needed, more slabs could be added to the planned scan.

The helicoidal scans were acquired with protocol standard, pitch 1, 3.2 mm slice width, 120 kVp, 200 mAs, filter B, FOV 500 mm, and an explored length to cover the whole length of the phantom and a matrix scan of 768×768.

### E. Informatics. Processing tools

Images were processed in a Merge eFilm 2.1 workstation (Merge Healthcare Inc., Mississauga, ON, Canada) of the PACS system, the software being ImageJ2x with an added Mtrack2 plug‐in (by N. Stuurman, 2003). Data obtained were processed with the software OriginPro 8.0724 (OriginLab Corp, Northampton, MA).

### F. Amplitude of the respiratory signal

**Figure 5:**
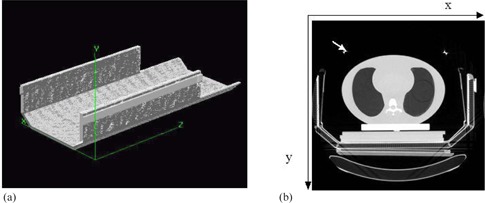
The stereotactic coordinate system (a), and the coordinate system (b) used in the ImageJ2x software, with the cine images.

**Figure 6:**
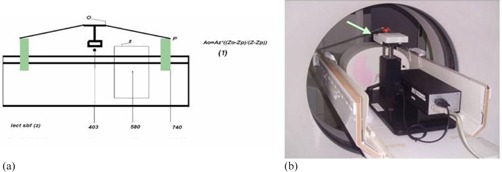
Schematic lateral view (a) of the system. Picture (b) of the system set up to obtain the CT images.

To process the images and to obtain the respiratory signal a coordinate system must be used. Figure 5(a) shows the built‐in coordinate system of the stereotactic body frame. The align procedure, with the laser of the CT and a level control tool, causes the DICOM coordinate (CT) system to be very similar in orientation but with a different origin to the stereotactic coordinate system. So the necessary Z readings $\left(Z,Z_{0},Z_{p}\right)$ can be read in any of the coordinate systems (DICOM or stereotactic coordinate system) to apply Eq. (1), these readings can be taken with a helicoidal CT scan that covers the whole length of the SBF from the central piece to the lateral pieces where the phantom is placed, from point O to point P (Fig. 6(a)).

Referring to the Y coordinate, the ImageJ2x software uses a coordinate system on the images where the origin is in the upper‐left corner of the image, as shown in Fig. 5(b). The coordinates of both wires obtained are processed to obtain the amplitude of their motion in that frame.

After detecting the Y coordinates of the wires in every frame of a slice. The amplitude of the motion of the wires in the slice can be calculated with Eq. (1).

(1) $$A_{z}=\frac{\left(y_{1max}-y_{1}+y_{2max}-y_{2}\right)}{2}$$

Here, $y_{1\text{max}}$ is the Y coordinate of the left wire detected on the cine images of a slice in a temporal frame where its position is closer to the patient table (exhale position), $y_{2\text{max}}$ is the Y coordinate of the left wire detected on the cine images of a slice in a temporal frame where its position is closer to the patient table (exhale position), $y_{1}$ is the Y coordinate of the left wire detected on the frame, $y_{2}$ is the Y coordinate of the right wire detected on the frame, and $A_{z}$ is the amplitude of the motion of the wires in any slice on a plane with coordinate Z.

A schematic lateral view (Fig. 6(a)) shows the system's setup and the necessary readings to process the information. Equation (2) shows how to calculate the amplitude of the vertical motion of the central piece.

(2) $$A_{0}=A_{z}\times \frac{\left(Z_{0}-Z_{p}\right)}{\left(Z-Z_{p}\right)}$$

where $A_{z}$ is the amplitude of the motion of the wires in any slice on a plane with coordinate Z, $Z_{0}$ is the Z coordinate of the central piece (point O in Fig. 6(a)), $Z_{p}$ is the $Z_{p}$ coordinate of the contact point of the rods with the pieces coupled to the lateral walls of the SBF (point P in Fig. 6(a)), and $A_{0}$ is the amplitude of the vertical motion of the central piece, as placed on a patient on a point between the umbilicus and the xiphoid process.

This equation calculates the amplitude of the vertical motion of the central piece. A robust indicator for the amplitude $A_{z}$ is the average of the amplitude of the motion of both wires seen on the CT images.

The distance from the platform (surrogate) to the CIRS phantom is longer and higher than the position of the diaphragm relative to the lungs of a regular‐sized patient. Therefore, the surrogate platform piece was changed by adding a Styrofoam piece that places the central piece of the system closer to the phantom, (see arrow in Fig. 6(b)).

### G. Tests performed

The base of the CIRS phantom was placed over two groups of three water‐equivalent slabs each 1 cm thick, inside the stereotactic body frame (SBF), the align procedure to obtain a CT acquisition with the SBF was performed^(9)^ and basically consisted of the following.

1. Position the patient in the frame, using the trunk and leg lasers.
2. Align the frame to the coordinate system of the CT scanner with the arc.
3. Align the frame to the horizontal plane with the level control.
4. Remove the arc, trunk and leg lasers.
5. Perform CT examination.

This procedure is described to align the patient (in the case of the phantom, the leg lasers were not used). The center of the phantom indicated by the external markers coincides with the longitudinal stereotactic coordinate Z, 580 mm, and the lateral coordinate X was 300 mm (center of the SBF).

Several CT studies were acquired. See Table 1 for details.

Different motions of the tumor and the surrogate were simulated in these studies; the motion of the surrogate was always correlated with the movement of the “tumor.” Spherical targets of 1 and 3 cm were used.

A respiratory cycle of 6 s was used because the time between images in the cine axial mode was always ∼0.8 s with a rotation time of 0.5 s. A cycle time of 3∼4 s or less will be better detected with the possibility of less time between images and a faster rotation time or a license to perform helicoidal studies with slow pitch values.

CIRS 10–12 were special studies where the amplitude of the movement of the surrogate was only ± 1 mm (11), ± 2 mm (10) and ± 3 mm (12) to check the sensitivity of the system to small movements of the central piece, equivalent to a patient with shallow breath.

Images were processed with the Merge eFilm workstation. Cine images corresponding to a slice position were extracted from every slab. In the most distal slab the cine images were extracted from the most proximal slice position to the central piece. It is more difficult to detect the amplitude of the respiratory signal in the slices of the most distal slab from the central piece. When the slice is more distal to the central piece, the amplitude of the movement of the wires, in the image, is smaller; therefore, checking these images with good results predicts better results in the rest of the images from the slabs that are closer to the central piece.

The proper detection of the respiratory signal in the proximal slice of the most distal slab to the central piece guarantees that at least in one slice of this slab the pulse can be detected, and also in the slices of the rest of the slabs.

The temporal images of these slices were exported and processed with ImageJ2x software, using mean and window tools, binary conversion, binary filters, and the Mtrack2 plugin, to obtain X and Y coordinates of the geometric center of the position of the wires in the image. Then the average amplitude of the motion of both wires was obtained (Eq. (1)), later the amplitude of the motion of the central piece of the system was obtained by applying Eq. (2). This respiratory signal then was processed with the Origin software to adjust the signal obtained to a sinusoidal function.

**Table 1:** CT studies performed to the phantom CIRS 008A placed on the SBF with the setup described.

*Study ID*	*Ct Acquisition Type*	*Motion Type*	*External Surrogate Motion*	*Internal Tumor Motion*	*Target Diameter*
CIRS01	helicoidal	No motion	NA	NA	Solid T 3 cm
		Sine wave T=6s	Sine wave ±10 mm	Long±10 mm	Solid T 3 cm
CIRS02	helicoidal			AP±5 mm	
				LL±5 mm	
		Sine wave T=6s	Sine wave ±10 mm	Long±10 mm	Solid T 3 cm
CIRS03	Cine 4 slabs			AP±5 mm	
				LL±5 mm	
		Sine wave T=6s	Sine wave ±6 mm	Long ±10 mm	Solid T 3 cm
CIRS04	Cine 4 slabs			AP±5 mm	
				LL±5 mm	
		Shark fin motion T=6s	Shark fin ±10 mm	Long ±10 mm	Solid T 3 cm
CIRS05	Cine 4 slabs			AP±5 mm	
				LL±5 mm	
CIRS06	helicoidal	No motion	NA	NA	Solid T 1 cm
		Sine wave T=6s	Sine wave ±10 mm	Long±10 mm	Solid T 1 cm
CIRS07	helicoidal			AP±5 mm	
				LL±5 mm	
	Cine 7 slabs	Sine wave T=6s	Sine wave ±10 mm	Long±10 mm	Solid T 1 cm
CIRS08	(the whole phantom)			AP±5 mm	
				LL±5 mm	
		Shark fin T=6s	Shark fin ±10 mm	Long±10 mm	Solid T 1 cm
CIRS09	Cine 4 slabs			AP±5 mm	
				LL±5 mm	
		Sine wave T=6s	Sine wave ±2 mm	Long±10 mm	Solid T 3 cm
CIRS10	Cine 4 slabs			AP±5 mm	
				LL±5 mm	
		Sine wave T=6s	Sine wave ±1 mm	Long±10 mm	Solid T 3 cm
CIRS11	Cine 4 slabs			AP±5 mm	
				LL±5 mm	
		Sine wave T=6s	Sine wave ±3 mm	Long±10 mm	Solid T 3 cm
CIRS12	Cine 4 slabs			AP±5 mm	
				LL±5 mm	

## III. RESULTS

### A. Respiratory signal

**Figure 7:**
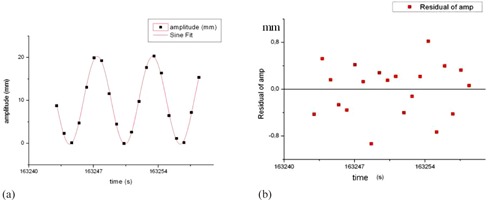
Fitted (a) and residual (b) amplitude for study CIRS003, CT slice 20 cm distant from the central piece.

The respiratory signal was obtained in all cine studies performed with the CIRS phantom. Results of the sine motion are shown in Table 2 and Fig. 7.

In Table 2 are shown the data of the fitting to a sine wave function (Eq. (3)) of the amplitude obtained on the processed cine images of a slice of the most distal slab from the different cine studies. The measured data are compared to the values of the function preset on the phantom to a sinusoidal function.

(3) $$y=y_{0}+A\times \text{sin}\left(\frac{2\pi (t-t_{c})}{T}\right)$$

where *A* is the amplitude of motion and *T* is the cycle time.

In Table 2 the results of the fits are shown for the period T of the motion of the central piece, and in Table 3 the results of the amplitude are shown. These results were obtained from a fit to a sine wave function of the amplitude obtained on the processed cine images of a slice of the most distal slab (>20 cm) from the different cine studies, and a comparison with the values of the function preset on the phantom.

In all cases the regression coefficient $R^{2}$ was > 0.97.

**Table 2:** Results of the period T from the measurements fitted to a sine wave function of the amplitude obtained on the processed cine images of a slice of the most distal slab from the different cine studies and a comparison with the values of the function preset on the phantom. Standard deviation was extracted from the residuals.

*Study*	*Preset T (s)*	*Measured T (s)*	*SD (s)*	*Temporal Discrepancy (s)*
CIRS03	6.00	5.983	0.017	‐0.017
CIRS04	6.00	6.001	0.014	+0.001
CIRS05	6.00	6.025	0.046	+0.025
CIRS08	6.00	5.998	0.200	‐0.002
CIRS09	6.00	6.027	0.092	+0.027
CIRS10	6.00	5.963	0.210	‐0.037
CIRS11	6.00	6.292	0.122	+0.292
CIRS12	6.00	5.991	0.017	‐0.009

**Figure 8:**
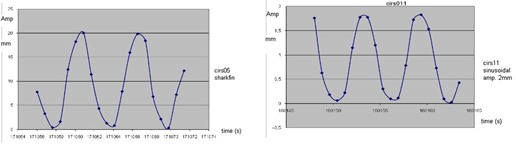
Amplitude (mm) vs. Time (s) graph (a), obtained in the studies CIRS05, 20 cm from the central piece (most distant slab) shark‐fin shape, amplitude ±10 mm (surrogate). Amplitude (mm) vs. Time (s) graph (b), obtained in the study CIRS11, 20 cm form the central piece (most distant slab) amplitude ±1 mm. These two studies were different from the others and most difficult to fit to a sine wave function, as is seen on the results of Table 2.

The studies where the regression coefficient value was <0.99 occurred in case CIRS 05, with an adjustment of a shark‐fin motion to a sine wave function; and in CIRS 11, where the preset amplitude of the central piece was ±1 mm. A graphic of the two cases is shown in Fig. 8.

**Table 3:** Results of the Amplitude A from the measurements fitted to a sine wave function of the amplitude obtained on the processed cine images of a slice of the most distal slab from the different cine studies and a comparison with the values of the function preset on the phantom. Standard deviation was extracted from the residuals.

*Study*	*Preset A (mm)*	*Measured A (mm)*	*SD (mm)*	*Amplitude Discrepancy (mm)*	*Adjusted* $R^{2}$	*Residual Max. (mm)*
CIRS03	10.00	10.24	0.16	+0.24	0.9954	0.817
CIRS04	6.00	6.19	0.08	+0.19	0.9969	0.580
CIRS05	10.00	9.98	0.36	‐0.02	0.9754	1.470
CIRS08	10.00	10.4	0.18	+0.40	0.9947	0.992
CIRS09	10.00	10.35	0.32	+0.35	0.9856	1.522
CIRS10	2.00	2.07	0.04	+0.07	0.9935	0.136
CIRS11	1.00	0.95	0.09	‐0.05	0.9757	0.170
CIRS12	3.00	3.09	0.04	+0.09	0.9953	0.250

## IV. DISCUSSION

In all respiratory signals obtained in the studies with sinusoidal motion the residuals of the fitted signal to the preset signal were within 1 mm.

The standard deviations (SD) calculated for the amplitudes were less than 0.4 mm in all cases. The differences in the preset amplitudes were less than 0.4 mm also.

Other methods, such as the Varian RPM method (Varian Medical Systems, Palo Alto, CA), provide an improved accuracy, with 0.04 mm SD from phantom motion reported.^(11)^ The described method has the following advantages:

1. Absolute measurement of the motion amplitude of the phantom or patient surrogate with sub‐millimeter accuracy.
2. Breathing phase information is imbedded in the CT image.
3. Simplicity and low cost.

The simplicity of this method, with small changes, suggests its use also as an add‐on to the phantom to visually check and assess the movement of the surrogate on the CT images. The system is designed for retrospective phase sorting of the images. It can also employ either amplitude or amplitude and velocity to bin the images.

With the current design it is impossible to obtain the images with prospective gating. This is a great disadvantage when compared to other methods. With a more complex approach and cooperation with manufacturers, prospective gating could be developed.

For example, a dedicated protocol in the CT equipment would permit:

- Additional image reconstruction with smaller FOV in the area of the wires for better detection of the respiratory signal.
- Automatic detection of the wire position in a reference slice, to start the acquisition prospectively in a selected phase with a short delay.
- A first reconstruction to detect the respiratory signal, interpolate this signal to the projection data, and then obtain a final reconstruction based on the data from the sinogram space.

In the treatment machine, a method to track the position of the central piece could be useful to trigger the treatment.

The system works best without abdominal compression. If compression is used, the central piece of the device should be attached to the patient using a custom elastic band, which is wrapped around the patient. This addition will guarantee the vertical motion of the central piece, the restriction of the respiratory movement, and the stabilization of the position of the central piece on the abdomen of the patient.

To obtain the 4D CT images with this method, the next step is to develop a software to bin the images and obtain the 3D image sets.

The proposed procedure allows radiotherapy services in LMIC to start performing simple 4D CTs for planning with minimal costs in hardware and software.

This device is simpler than other reported methods. Other methods rely on complex equipment and require a proper synchronization; on the other hand, deviceless methods require complex postprocessing and are not always easy to evaluate.

It is possible to see clearly the displacement of the wires reviewing the whole set of cine images in a single slice (when the amplitude of the motion of the vertical piece is ~ 3 mm or bigger).

This device can be seen by the patient. A cooperative patient can use this visibility to assist with coached breathing. Also notable about this system is that some points of these bars, at some distance from the central piece, also have longitudinal motion. Therefore it might be possible to obtain projections in cone‐beam CT and in PET‐CT that will also have the information of the respiratory signal. Something similar to CT occurs in MRI if the bars, instead of having a wire inside, have a solution of copper sulfate.

## V. CONCLUSIONS

A simple and low‐cost method was has been described to for obtaining a respiratory signal in a dynamic phantom with good precision and coincidence with the preset motion.

This method can be adapted, with small changes, to other systems of immobilization and positioning of the patients to be treated with SBRT. However, the use of a stereotactic body frame is recommended.
